# A wonderful network unraveled - Detailed description of capillaries in the prosomal ganglion of scorpions

**DOI:** 10.1186/1742-9994-11-28

**Published:** 2014-05-09

**Authors:** Bastian J Klußmann-Fricke, Sebastian W Pomrehn, Christian S Wirkner

**Affiliations:** 1 Allgemeine & Spezielle Zoologie, Institut für Biowissenschaften, Universität Rostock, Universitätsplatz 2, 18055 Rostock, Germany

## Abstract

**Introduction:**

Though it has long been known that the prosomal ganglion of scorpions is supplied by a dense system of arteries, the pattern of this network has never been described and analyzed in detail. Using MicroCT in combination with computer aided 3D-reconstruction we provide the first detailed description of the pattern of arteries in the prosomal ganglion of *Brotheas granulatus* (Scorpiones, Chactidae) and other scorpion species.

**Results:**

The entire prosomal ganglion in scorpions is supplied by a network of arteries that branch off the major arteries of the anterior aorta system. The most prominent of these are the nine transganglionic arteries which run through the nerve mass along the midline of the body and branch terminally, i.e. below the neuropils, into smaller arteries. These arteries reticulate into a dense network between the surrounding somata and the centrally located neuropil structures of the ganglion.

**Conclusions:**

We demonstrate the presence in the prosomal ganglion of scorpions of a capillary system made up of afferent arteries which deliver hemolymph into the ganglion and efferent arteries which transport the hemolymph out of the ganglion. Adopting the structural definition used for vertebrate circulatory systems, this capillary network can also be termed a *bipolar rete mirabile* (located as it is between afferent and efferent arteries) analogous to those found in vertebrates and some echinoderms.

Within the *rete mirabile* of the scorpion prosomal ganglion, some regions (i.e. neuropils) are better supplied than others. The structural information provided here can now be used in functional neuronal studies to determine the physiological and computational significance of the various neuropils in the complex scorpion nervous system.

## Introduction

The central nervous system (CNS) is one of the most important organ systems in animals, so ensuring that it is supplied with nutrients and oxygen is a crucial matter. In arthropods, the central nervous system consists of a 'syncerebrum' and a number of free segmental ganglia which are situated ventrally in the body and therefore known as the ventral nerve cord [1]. In arachnids, the syncerebrum is fused with a taxon-specific number of ventral nerve cord ganglia to form the condensed prosomal ganglion (Pg; [2]). A prosomal ganglion combines a dorsal supra- and a ventral subesophageal ganglia. Classically, supra- and subesophageal ganglia are connected by circumesophageal connectives [3], but these are not distinguishable in arachnids [2], where the supraesophageal ganglion sits broadly on the anterior part of the subesophageal ganglion. Only in lateral view can the rather thin esophagus be taken as a fictive border between the two major ganglionic masses.

**Figure 1:**
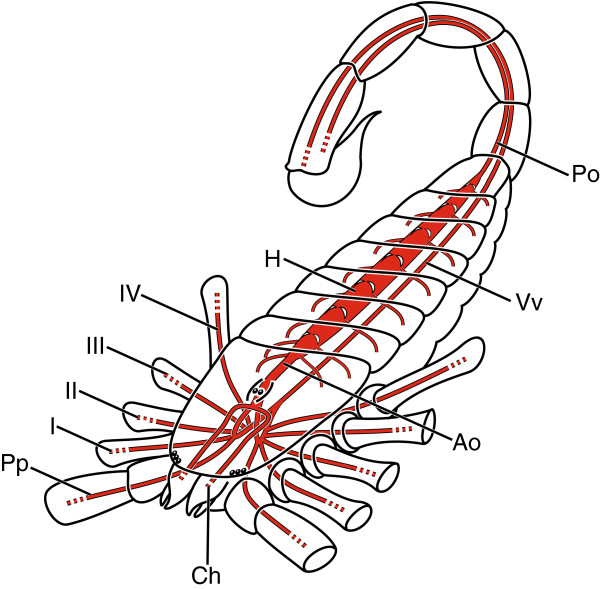
**Schematic drawing of major vascular system of Scorpiones (modified from **[6]** with permission from Wiley and Son).** Abbreviations: Ao, anterior aorta; Ch, cheliceral arteries; H, heart; I-IV, leg arteries; Po, posterior aorta; Pp, pedipalpal artery; Vv, ventral vessel.

The prosomal ganglion is supplied by the hemolymph vascular system (HVS), which in scorpions consists of a dorsal tubular heart located in the midline of the mesosoma (Figure 1; H) and surrounded by a sac-like pericardium [2,4-6]. Hearts are equipped with seven pairs of ostia and six or eight pairs of cardiac arteries which supply the organs of the mesosoma. Hearts are connected posteriorly to the posterior aorta, which runs dorsally through the metasoma into the telson (Figure 1; Po), and anteriorly to the anterior aorta, which runs into the prosoma to the level of the supraesophageal ganglion and goes on to supply, via a complex branching pattern, the various organ systems and appendages (Figure 1; Ao).

It has long been known that the prosomal ganglion in scorpions is supplied by a number of vessels [4,7-9], and even that these vessels form a dense network [2,6]. However, the structure and pattern of the network has never been studied in detail. At first glance, the fine arteries in question could be interpreted as capillaries, which in vertebrates are defined as networks of small-diameter vessels (5 – 10 μm) that connect arteries and veins. Capillaries are characterized by the presence of exclusively endothelial layers and connective tissue, and are where the exchange of oxygen and nutrients takes place [10]. As veins are not present in arthropods [11], the older literature on arthropods (or more precisely on crustaceans) defines capillaries either as small-caliber vessels continuous with the efferent arteries and without visible intima [12], or as vessel networks that link afferent and efferent vessels [13].

This paper provides the first detailed description of the morphology of the cerebral vasculature in scorpions. It also identifies parts of the prosomal ganglion that are better supplied than others and discusses possible reasons for this pattern. We discuss the terminology commonly used to describe capillaries and *retia mirabilia* and the possible adoption of these terms to arthropod circulatory systems in general. Furthermore, we hypothesize on how the pattern of arteries described might be established during ontogeny.

## Results

### Gross morphology of the prosomal ganglion and the vascular system which supplies it

**Figure 2:**
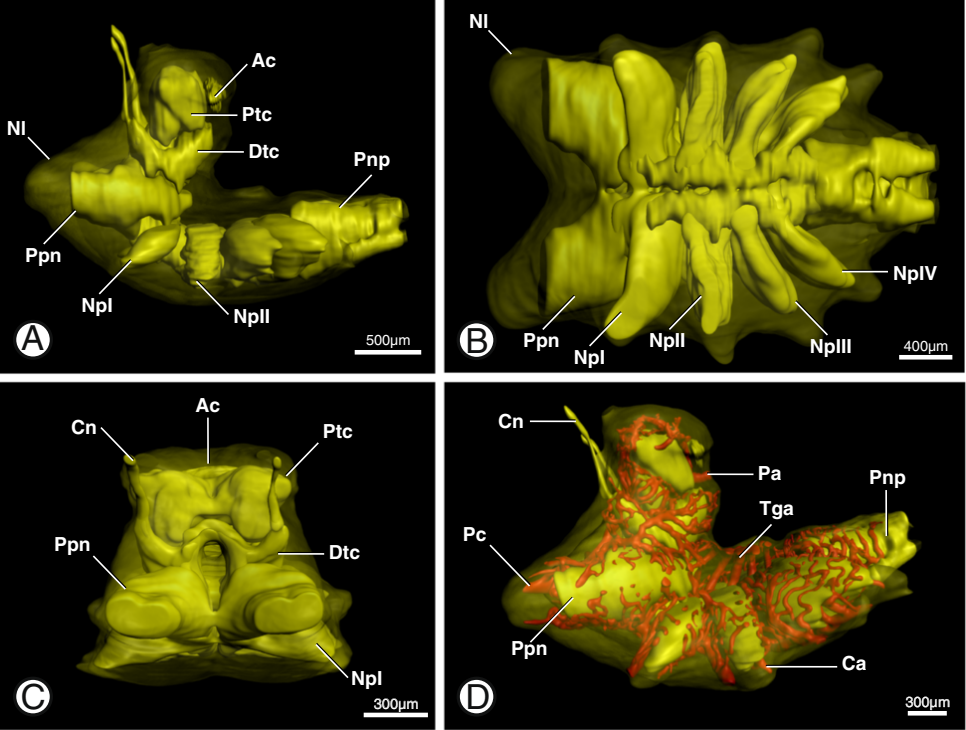
**Gross morphology of the prosomal ganglion in *B. granulatus*. A**: Surface renderings of the neurilemma (Nl; transparent) and the neuropil of the prosomal ganglion showing the general organization of the ganglion; note that space between neurilemma and neuropils is filled with somata (not shown); in lateral view. **B**: Surface renderings of the neurilemma (Nl) and the neuropil of the prosomal ganglion showing pedipalpal (Pnp) and leg neuropils (NpI-IV); in ventral view. **C**: Surface renderings of the neurilemma and the neuropil of the prosomal ganglion showing neuropils of the proto- (Ptc), deuto- (Dtc), and tritocerebrum (i.e. pedipalpal neuromere; Ppn); in anterior view. **D**: Surface renderings of the neurilemma, neuropils and cerebral vasculature of the prosomal ganglion giving an overview of the ganglionic vessels; in lateral view. Abbreviations: Ac, arcuate body; Cn, cheliceral nerve; Ca, coxal arteries; Dtc, deutocerebral neuropil; Nl, neurilemma; NpI-IV, leg neuropil I-IV; Pa, protocerebral artery; Pc, pedipalpocoxal arteries; Pnp, posterior neuropil; Ppn, pedipalpal neuropil; Ptc, protocerebral neuropil; Tga, transganglionic arteries.

**Figure 3:**
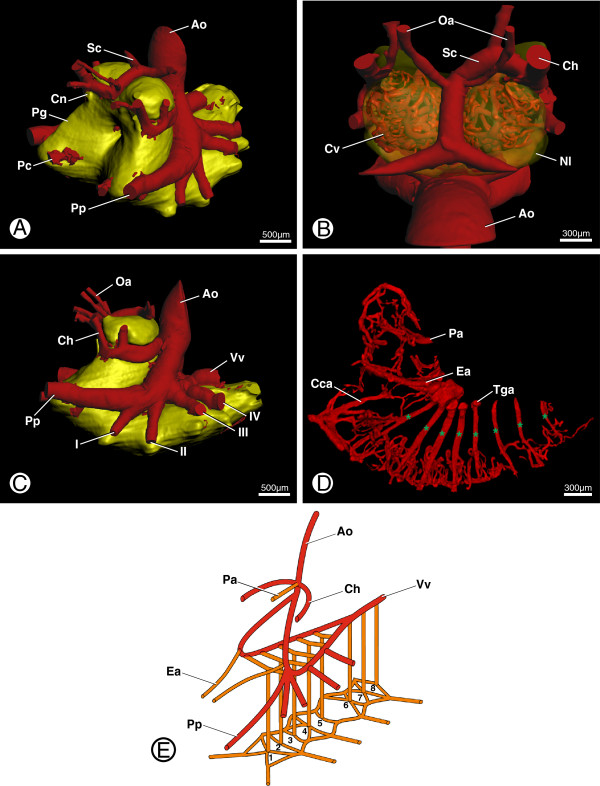
**Afferent hemolymph supply to the prosomal ganglion in *B. granulatus*.** The PDF version of this article contains interactive 3D content. Please click in the file on figure **A** - **D** to activate the content and then use the mouse to rotate the objects. Use the menu in the activated figure to use further functions. **A**: Surface renderings of the anterior aorta system and the prosomal ganglion (Pg; yellow) showing general branching pattern of anterior aorta system; in anterolateral view. **B**: Surface renderings of the anterior aorta (Ao), branching arteries (e.g. cheliceral arteries, Ch), and the cerebral vasculature (Cv); in dorsal view. **C**: Surface renderings of the anterior aorta system and the prosomal ganglion (Pg; yellow) showing general branching pattern of prosomal arteries; in lateral view. **D**: Volume rendering of the ganglionic midline arteries in lateral view; asterisks indicating the eight transganglionic arteries (Tga); in lateral view. **E**: Schematic drawing of the prosomal vascular system (red) and the main afferent arteries (orange) of the prosomal ganglion; numbers denote transganglionic arteries 1-8. Abbreviations: Ao, anterior aorta; Cca, central collection artery; Ch, cheliceral arteries; Cn, cheliceral nerve; Cv, cerebral vasculature; Ea, esophageal artery; I-IV, leg arteries; Nl, neurilemma; Oa, optic arteries; Pa, protocerebral artery; Pc, pedipalpocoxal arteries; Pg, prosomal ganglion; Pp, pedipalpal arteries; Sc, supracerebral artery; Tga, transganglionic arteries; Vv, ventral vessel.

In the following, morphology of the prosomal ganglion and the vascular system which supplies it is described for *Brotheas granulatus*. Comparative morphological aspects within scorpions are described in a separate chapter below. The terminology used in the following description of the prosomal ganglion follows the works of Babu [14] and Horn and Achaval [3]. As stated above, the prosomal ganglion comprises a supra- and a subesophageal ganglion. Its nervous tissue consists of densely packed peripheral somata (Figure 2, space between neurilemma – Nl and neuropil) and centrally located neuropil structures (e.g. Figure 2; NpI) made up of the axons and synapses of the neurons. The supraesophageal ganglion consists of the posterior arcuate body (see below), with the remaining protocerebrum receiving the optic nerves and the deutocerebrum, i.e. the cheliceral neuromere (Figure 2A, B; Ptc + Dtc). The arcuate body (*sensu* Loesel et al. [15]) (Figures 2A, C and 3B; Ac) is an unpaired double-layered midline neuropil which is located at the posterior border of the protocerebrum. The subesophageal ganglion is formed by the neuromeres of the pedipalps (i.e. the tritocerebrum) (Figure 2; Ppn), the neuromeres of the four pairs of limbs (Figure 2; NpI-IV), and four incorporated ganglia of the ventral nerve cord (Figure 2; Pnp). The latter are known as the posterior neuropils (*sensu* Babu, [14]) and include the pectinate neuropils (see [16] for details). In the anteroventral midline of the subesophageal ganglion laterally to the first five transganglionic arteries (see below), the paired central ganglion (*sensu* Babu, [14]) can be found. The whole prosomal ganglion is surrounded by the multilayered neurilemma (Figures 2A, B, and 7A; Nl).

From its origin at the heart, the anterior aorta (Figure 1; Ao) runs in an anteroventral direction to the posterodorsal part of the supraesophageal ganglion (Figure 3A-C; Ao) where the cheliceral arteries branch off and run around the supraesophageal ganglion laterally (Figure 3B, C; Ch). Anterior to the supraesophageal ganglion these arteries bend upwards and run into the chelicerae (Figure 3C). Shortly after this bend, a supracerebral artery (Figure 3B; Sc) emanates either from the left or the right cheliceral artery (see [2] for details on variation).

After the cheliceral arteries have branched off dorsally to the thin esophagus, the anterior aorta bifurcates into two lateral trunks from which subsequently the arteries of the pedipalps and the four pairs of walking legs branch off (Figure 3C; Pp, I-IV). On top of the subesophageal ganglion, the lateral trunks curve backwards, reunite, and give rise to the ventral vessel (Figure 3C; Vv). This artery runs atop the ventral nerve cord in a posterior direction through the entire opisthosoma, supplying the free ganglia of the nerve cord.

### Afferent systems of the prosomal ganglion

The prosomal ganglion is exclusively supplied by arteries which emanate from the anterior aorta system. The most prominent of these are the nervous midline arteries, which emanate from the aortic trunks. The midline arteries can be broken down into the protocerebral artery (Pa) and the paired esophageal arteries (Ea) in the supraesophageal ganglion, and the transganglionic arteries (Tga) in the subesophageal ganglion (Figure 3D, E). Wherever they come into direct contact with the prosomal ganglion, the arterial subsystems of the anterior aorta (e.g. the cheliceral arteries and the leg arteries) give rise to many other small arteries too (Figure 5C, D).

The first midline artery, i.e. the protocerebral artery, runs in an anterior direction through the prosomal ganglion while the last, i.e. the 8^th^ transganglionic artery (Figure 3D; 8^th^ asterisk), emanates in a ventral direction. The 9^th^ transganglionic artery is a special case since it does not run through the subesophageal ganglion but emanates from the ventral vessel at the posterior border of the prosomal ganglion (not shown). This relatively thick artery runs in a ventral direction and branches terminally into one anterior and one posterior artery. The former anastomoses with the 8^th^ transganglionic artery and the latter splits after a short distance and gives rise to the pectinate arteries.

### Supraesophageal ganglion supply

**Figure 4:**
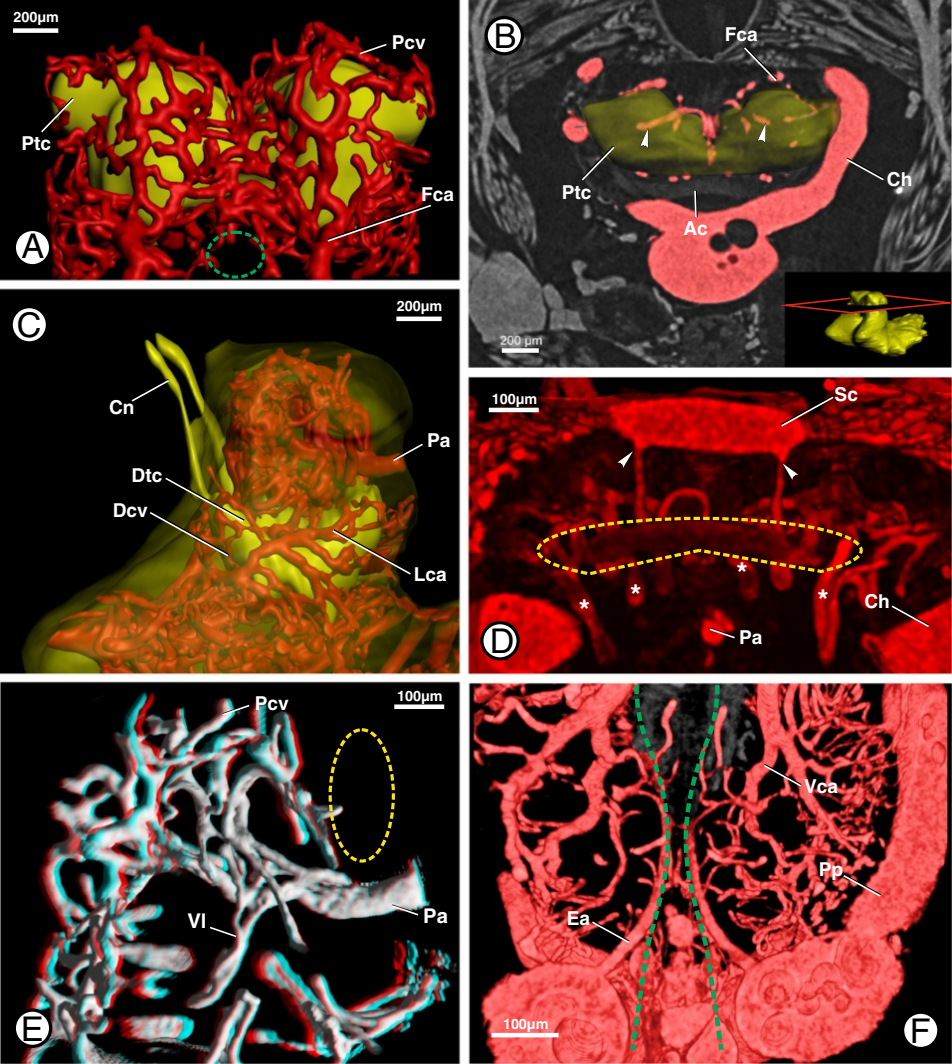
**Hemolymph supply to the protocerebrum (Ptc) and deutocerebrum (i.e. cheliceral neuromere; Dtc) in B. granulatus. A**: Surface renderings of the protocerebral neuropil (Ptc) and protocerebral vessels (Pcv) showing general pattern of arteries in the protocerebrum; in anterior view; dashed green line indicates the position where the esophagus pierces the prosomal ganglion. **B**: Virtual horizontal section through the protocerebrum with a transparent surface rendering of the protocerebral neuropil (Ptc); in dorsal view; inset shows the position of the section, arrow heads indicate vessel loops that protrude into the neuropil. **C**: Surface renderings of the neurilemma, deutocerebral neuropil (Dtc) and deutocerebral vasculature (Dcv); in lateral view. **D:** Volume rendering of the protocerebral vasculature showing supply to the arcuate body; in posterior view; dashed yellow line indicates the position of the arcuate body, asterisks indicate arteries supplying the arcuate body, arrowheads indicate two arteries that emanate from the supracerebral artery (Sc). **E:** 3D image (use 3D glasses) of a volume rendering of the protocerebral artery (Pa) showing a vessel loop (Vl) protruding into the neuropil; in lateral view; dashed yellow line indicates the position of the arcuate body. **F:** Volume rendering of the cerebral vasculature showing the origin of the esophageal arteries (Ea); in dorsal view; dashed green lines indicate the position where the esophagus passes the prosomal ganglion. Abbreviations: Ac, arcuate body; Ch, cheliceral arteries; Cn, cheliceral nerves; Dcv, deutocerebral vasculature; Dtc, deutocerebral neuropil; Ea, esophageal arteries; Fca, frontal collection artery; Lca, lateral collection artery; Pa, protocerebral artery; Pcv, protocerebral vasculature; Pp, pedipalpal arteries; Ptc, protocerebral neuropil; Sc, supracerebral artery; Vca, ventral collection artery; Vl, vessel loop.

The supraesophageal ganglion is mainly supplied by the protocerebral artery, the esophageal arteries and a number of small arteries which emanate from the cheliceral arteries. The protocerebral artery emanates directly from the anterior aorta between the cheliceral arteries (Figure 3D; Pa). It curves slightly ventrally and then runs beneath the arcuate body in an anterior direction into the neuropil of the remaining protocerebrum (Figure 4D, E). After a short distance, the protocerebral artery splits into two smaller arteries. The dorsal branch (i.e. the thinner one) bends dorsally, where it splits and ramifies into smaller arteries. The latter bend laterally, forming loops within the protocerebral neuropil, and anastomose with surrounding arteries (Figure 4A, E; Pcv). The ventral branch of the protocerebral artery runs further in an anterior direction to the border of the protocerebral neuropil, where it branches into smaller reticulating arteries (Figure 4D). These arteries form a number of anastomoses thus forming a network of arteries located directly on the border between the neuropil and the surrounding somata (Figure 4A; Ptc). From various points of the arterial network single arteries run into the neuropil of the protocerebrum, each forming a twisted loop and running back into the network (Figure 4B; arrowheads). Twisted loops of this kind can be found in almost all neuropils.

The arcuate body is mainly supplied by a number of ventrally oriented arterial loops (Figure 4D; asterisks) which emanate from the network of arteries within and around the protocerebrum. In addition, there are two small arteries which emanate from the supracerebral artery a short distance after its spilt (see above, Figure 3B). These two arteries run ventrally, then also form loops and anastomoses with the other arteries that supply the arcuate body (Figure 4D; arrowheads).

The cheliceral neuromere is mainly supplied by the esophageal arteries, the only paired midline arteries. They emanate from the lateral trunks of the anterior aorta (Figure 4F; Ea) laterally to the esophagus (Figure 4F; dashed green lines), at the point at which the latter passes through the prosomal ganglion. Each of the esophageal arteries runs in an anterior direction alongside the esophagus, ramifying distally into smaller arteries which anastomose with the surrounding cerebral arteries. Additionally, small arteries run ventrally to supply the pedipalpal neuropil (see below). As described for the protocerebrum, the arteries reticulate directly at the border between the neuropil and the surrounding somata, also forming loops that protrude into the neuropil.

At the anterior border of the neuropils, the arteries supplying the corresponding neuromeres unite to form two laterally positioned frontal collecting arteries (Figure 4A, B; Fca) which run in a ventral direction before finally discharging into the pedipalpocoxal arteries (see below).

### Subesophageal ganglion supply

The subesophageal ganglion is mainly supplied by the transganglionic arteries and the small arteries which emanate from the pedipalpal arteries and leg arteries.

**Figure 5:**
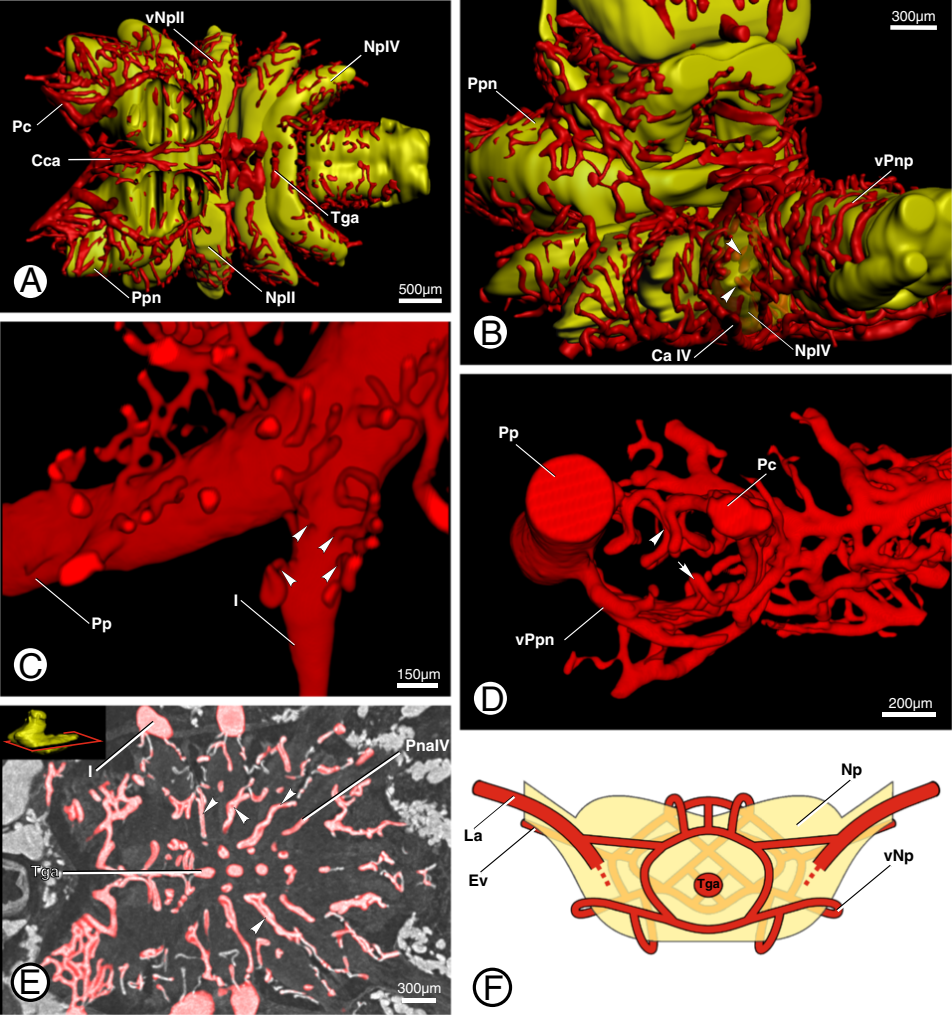
**Supply to specific neuropils in *B. granulatus*. A**: Surface renderings of the ventral cerebral vasculature and the neuropils of ventral neuromeres in the prosomal ganglion give an overview of the supply system of the subesophageal ganglion; in dorsal view. **B**: Surface renderings of the posterior cerebral vasculature and the neuropils of posterior neuromeres in the prosomal ganglion showing supply to the neuropil of the leg neuromere IV (NpIV); in posterolateral view; arrowheads indicate vessel loops that protrude into the neuropil. **C**: Volume rendering of supplying arteries emanating from the left pedipalpal artery (Pp) and leg artery I (I); in ventrolateral view; arrowheads indicate arteries that emanate from the appendage artery. **D**: Volume rendering of arteries supplying the pedipalpal neuropil (vPpn) showing vessel loops that protrude into the neuropil; in anterior view; arrowhead indicates larger, dorsal vessel loops, arrows indicate smaller, ventral vessel loops. **E**: Virtual horizontal section through ventral parts of the prosomal ganglion showing the postneuropilar arteries (PnaIV); in dorsal view; inset shows the position of the section, arrowheads indicate the postneuropilar arteries. **F**: Schematic drawing of the supply system of an isolated neuropil showing the network of supplying arteries (vNp); in dorsal view. Abbreviations: CaIV, coxal artery IV; Cca, central collection artery; Ev, efferent vessel; La, leg artery; Np, neuropil; NpII + IV, neuropil of leg II + IV neuromere; Pc, pedipalpocoxal arteries; PnaIV, postneuropilar artery of leg IV neuromere; Pp, pedipalpal artery; Ppn, neuropile of pedipalpal neuromere; Tga, transganglionic artery; vNp, vessels of a certain neuropil; vPnp, vessels of posterior neuropil; vPpn, vessels of pedipalpal neuropil.

The neuropils of the pedipalps and walking legs are surrounded by dense networks of arteries which, in the main, emanate from the arteries of the corresponding appendage at the area at which the latter make contact with the subesophageal ganglion (Figure 5C, D). From each appendage artery (e.g. the pedipalpal artery), two rows of smaller arteries emanate which reticulate around the border of the corresponding hemi-neuromere (Figure 5C; arrowheads). Posteroventrally of each of the leg arteries a postneuropilar artery emanates, running along the posterior surface of the corresponding hemi-neuromere in the direction of the transganglionic arteries (Figure 5E; Pna IV and arrowheads). Just before it reaches the transganglionic arteries, the postneuropilar artery forms a loop (Figure 5E; arrowheads), runs a short distance backwards along its previous course and then discharges into the arterial network that surrounds the hemi-neuromere. From these arterial networks, loops run into the neuropil (Figure 5B; arrowheads). Additionally, in the vicinity of the pedipalpal hemi-neuromeres, much smaller arterial loops run into the neuropil from the ventral surrounding vasculature (Figure 5D; arrow). After encircling the neuropil, the arteries discharge into the corresponding collecting artery, which then leaves the ganglion.

**Figure 6:**
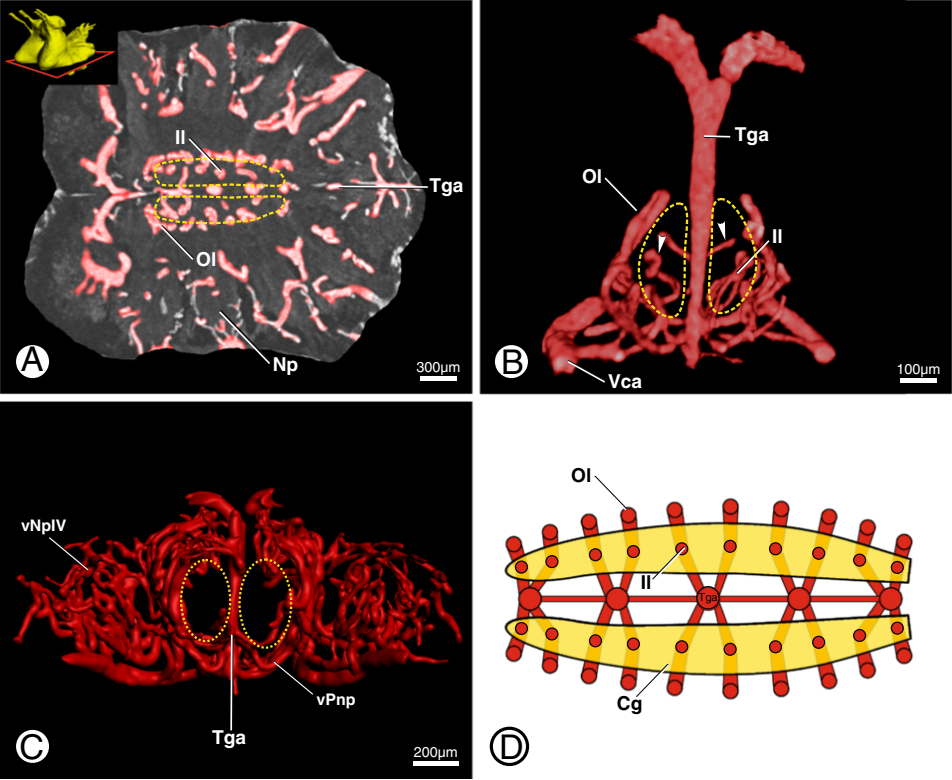
**Supply to the central ganglion and posterior neuropils in *B. granulatus*. A**: Virtual horizontal section through anteroventral parts of the ventral prosomal ganglion showing supply to the central ganglion; in dorsal view; inset shows the position of the section, dashed yellow lines show position of the central ganglion. **B**: Volume rendering of one transganglionic artery (Tga) and emanating arteries; in anterior view; dashed yellow lines indicate the position of the central ganglion, arrowheads indicate the inner row of vessel loops (Il) protruding into the central ganglion. **C**: Surface renderings of the vasculature of the posteroventral prosomal ganglion showing hemolymph supply to the posterior neuropil; in posterior view; dotted yellow lines show position of the posterior neuropil. **D**: Schematic drawing of the supply system of the central ganglion showing the inner (Il) and outer (Ol) row of vessel loops; in dorsal view. Abbreviations: Cg, central ganglion; Il, inner loop; Np, neuropil; Ol, outer loop; Tga, transganglionic artery; Vca, ventral collection artery; vNpIV, vessels of neuropil of leg IV neuromere; vPnp, vessels of posterior neuropil.

Once they have pervaded the subesophageal ganglion, transganglionic arteries 1 to 5 branch into smaller arteries which curve dorsally, forming a more medial inner and a more lateral outer row of loops around the central ganglion (*sensu* Babu, [14]) (Figure 6B; Ol + Il). The inner row forms smaller loops and runs around the tracts of the central ganglion while the outer row forms larger loops and runs around the border of the central ganglion (Figure 6A, B, D; Cg or dashed yellow line). Neighboring transganglionic arteries are connected with each other by at least one small artery.

The posterior neuropil is mainly supplied by the last three transganglionic arteries, which emanate from the ventral vessel. These arteries also branch terminally and reticulate on the ventral surface of the posterior neuropil (Figure 6C; dotted yellow line). Two additional rows of arteries that emanate from the ventral vessel laterally to the transganglionic arteries surround the borders of the posterior neuropil. There are fewer loops in the posterior neuropil than in the anterior neuromeres (Figure 6C). Furthermore, the pectinate neuropils were not observed to have their own supply.

### Efferent arteries

**Figure 7:**
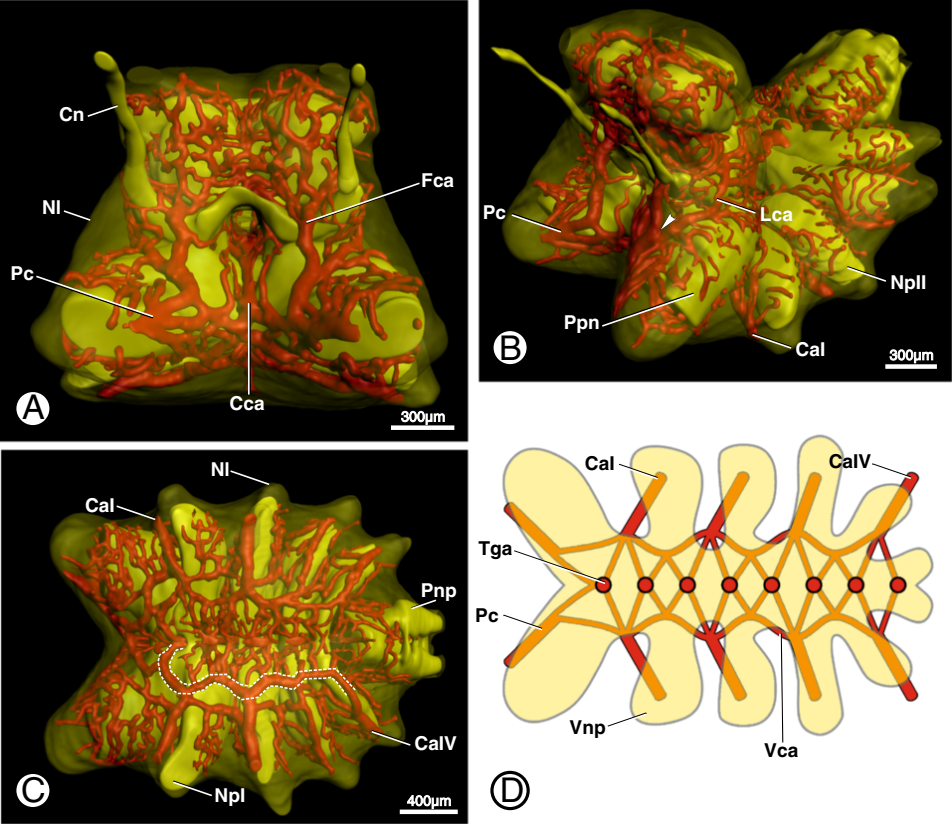
**Efferent arteries of the prosomal ganglion in *B. granulatus*. A**: Surface renderings of the neurilemma (Nl, transparent), the cerebral vasculature, and certain neuropils showing the frontal collection artery (Fca) and the pedipalpocoxal arteries (Pc); in anterior view. **B**: Surface renderings of the neurilemma (Nl), the cerebral vasculature, and certain neuropils showing the lateral collection artery (Lca); in anterolateral view; arrowhead indicates the position where the lateral collection artery (Lca) enters the frontal collection artery. **C**: Surface renderings of the neurilemma (Nl), the cerebral vasculature, and certain neuropils showing the ventral collection arteries (dashed white lines) and the efferent coxal arteries (CaI + IV); in ventral view. **D**: Schematic drawing of the ventral collection arteries (Vca), ventral neuropils (Vnp), and ventral efferent vessels; in dorsal view. Abbreviations: CaI, coxapophysal artery I; CaIV, coxal artery IV; Cca, central collection artery; Cn, cheliceral nerve; Fca, frontal collection artery; Lca, lateral collection artery; Nl, neurilemma; NpI + II, neuropil of leg I + II neuromere; Pc, pedipalpocoxal arteries; Pnp, posterior neuropils; Ppn, neuropil of pedipalpal neuromere; Tga, transganglionic artery; Vca, ventral collection arteries; Vnp, ventral neuropils.

All described arteries in the prosomal ganglion empty into a number of collection arteries through which the hemolymph leaves the ganglion. The most prominent of these efferent arteries are the pedipalpocoxal arteries (Figures 2D, 3A, 4A and 7A, B + D; Pc), which leave the prosomal ganglion medially to the pedipalpal arteries and run anterolaterally into the coxae of the pedipalps. The hemolymph which leaves the prosomal ganglion through the pedipalpocoxal arteries has thus passed through the ganglion mainly via the arterial networks around the neuropils of the proto-, deuto- and, tritocerebrum. In the proto- and deutocerebrum, a frontal collection artery (Fca) was observed in the midline of each hemi-neuromere running in a ventral direction (Figures 4A-C and 7A; Fca) and discharging into the pedipalpocoxal arteries. Another comparatively thick artery discharges into the frontal collection arteries (Figure 4C; Lca). The lateral collection arteries emanate posterolaterally from between the neuropils of the deuto- and tritocerebrum (Figures 4C and 7B; Lca). They run around the borders of the tritocerebrum and discharge into the frontal collection arteries on top of the pedipalpal neuropil (Figure 7B; arrowhead). Another collection artery could be distinguished ventrally of the point where the esophagus enters the prosomal ganglion in the midline of the anterior prosomal ganglion (Figures 5A and 7A; Cca). This central collection artery runs through the cleft between the neuropils of the pedipalpal hemi-neuromeres in an anteroventral direction, discharging into the frontal collection arteries.

The ventral collection arteries (Figure 7C; white dashed lines) are situated between the border of the subesophageal neuropils and the ventral cluster of somata, forming a network with numerous interconnecting smaller arteries (Figure 7D; Vca). The main collection arteries run in an undulating course the whole length of the subesophageal ganglion, giving rise to four pairs of coxal arteries which leave the ganglion ventrally of each hemi-neuromere of the corresponding leg neuromere (Figure 7B, C; CaI + IV). The first two pairs of coxal arteries are the coxapophysal arteries, which supply the maxillary glands (see [2] for details). The maxillary glands are located in the coxapophyses of the first two pairs of walking legs.

### Comparative morphological aspects within scorpions

**Figure 8:**
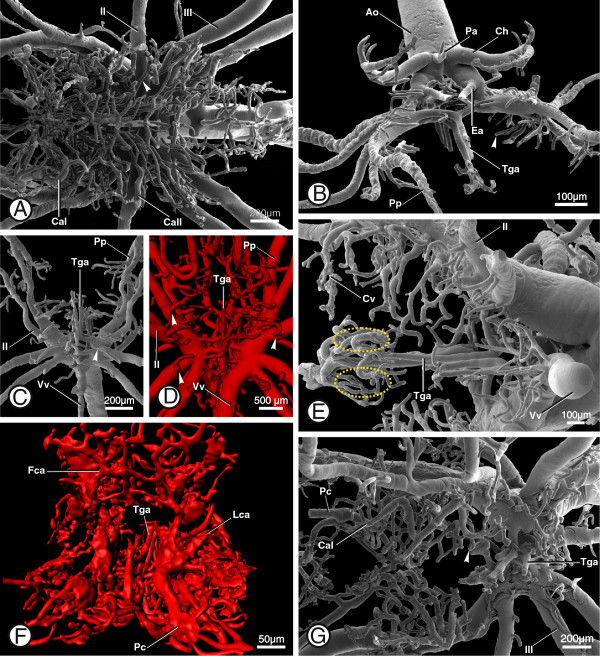
**Various aspects of the cerebral vasculature in different scorpion taxa. A**: SEM of a corrosion cast of the cerebral vascular system in *Lychas mucronatus*, Buthidae; ventral view; overview of the cerebral vasculature; showing coxal arteries (Ca1, 2), arrowhead indicates postneuropilar arteries. **B**: SEM of a corrosion cast of the prosomal vascular system in *Serradigitus joshuaensis*, Vaejovide; anterior view; showing midline arteries (Tga, Ea, Pa), arrowhead indicating a vessel loop. **C**: SEM of a corrosion cast of the prosomal vascular system in *Superstitionia donensis*, Superstitioniidae; ventral view; showing transganglionic arteries (Tga), arrowhead indicates a postneuropilar artery forming a vessel loop. **D**: Volume rendering of the prosomal vascular system in *Hadrurus arizoniensis*, Iuridae; ventral view; showing transganglionic arteries (Tga); arrowheads indicate postneuropilar arteries. **E**: SEM of a corrosion cast of the cerebral vascular system in *Diplocentrus lindo*, Diplocentridae; posterior view, ventral is left; showing transganglionic arteries (Tga), dashed yellow lines indicate the position of the central ganglion. **F**: Surface renderings of the cerebral vasculature in *Bothriurus keyserlingi*, Bothriuridae; anterior view; showing efferent arteries (e.g. Lca, lateral collection artery). **G**: SEM of a corrosion cast of the cerebral vascular system in *Uroctonus mordax*, Vaejovidae; ventral view; showing efferent arteries (e.g. Pc, pedipalpocoxal arteries), arrowhead indicates a vessel loop. Abbreviations: Ao, anterior aorta; Ca I + II, coxapophysal arteries; Ch, cheliceral arteries; Cv, cerebral vasculature; Ea, esophageal arteries; Fca, frontal collection artery; II + III, legarteries; Lca, lateral collection arteries; Pa, protocerebral artery, Pc, pedipalpocoxal arteries; Pp, pedipalpal arteries; Tga, transganglionic arteries; Vv, ventral vessel.

For comparison we also studied the cerebral vasculature of other species from different scorpion taxa (see taxon sampling in [2]). In all studied scorpion species, the prosomal ganglion is supplied by a dense network of arteries. Capillarization featuring afferent (e.g. the transganglionic arteries; Tga) and efferent arteries (e.g. the coxal arteries; Ca) was observed in all studied species (Figure 8A - G). We also observed the presence of vessel loops even in very small scorpion species (e.g. *Superstitionia donensis*, Superstitioniidae; Figure 8C; arrowhead).

The protocerebral arteries, the paired esophageal arteries and the nine transganglionic arteries which branch terminally into smaller arteries were found in all studied taxa (Figure 8B; Pa, Ea; Tga). Neighboring transganglionic arteries are interconnected. The arteries that supply the neuromeres of the prosomal ganglion also reticulate around the borders of the neuropil structures, sending twisted loops between the nerve fibers (Figure 8B, C, G; arrowheads). The postneuropilar arteries of the limb neuromeres are present in all studied scorpion species (Figure 8A, C, D; arrowheads). Aside from the transganglionic arteries, the most prominent arteries to run into the prosomal ganglion are those emanating posterolaterally from the leg arteries. We also observed that the degree of vascularization of certain parts of the ganglion (e.g. the central ganglion, see above) is higher than in others (Figure 8E; dashed yellow line).

## Discussion

### Comparative morphological aspects

The arterial patterns described above with a capillarization in the prosomal ganglion, protocerebral arteries, the paired esophageal arteries and the nine transganglionic arteries and the described efferent arteries can be hypothesized to belong to the ground pattern in scorpions. They may even represent apomorphies for scorpions, but this would need to be tested further using out-groups with complex arterial systems, such as tetrapulmonates (i.e. spiders and their close relatives; see below).

We did, however, also find some taxon-specific differences in the pattern of arteries which supply the prosomal ganglion. In Buthidae, for example, the cerebral vasculature is not as dense as in *B. granulatus*, and the arteries within the prosomal ganglion are not as highly organized (Figure 8A). There are some other differences in the cerebral vasculature of the different scorpion taxa which may provide further morphological characters for phylogenetic analysis (see [2]).

Within ‘ pulmonate’ arachnids, Araneae possess similarly complex arterial systems [17]. E.g. in the spider *Cupiennius salei* (Ctenidae), the supraesophageal neuromeres are supplied bilaterally by ventral branches of the cheliceral artery, arteries which split off from the two branches of the anterior aorta, and by arteries which branch off the first leg artery [18]. The arcuate body is supplied anteriorly by arteries emanating from the cheliceral arteries which enclose the arcuate body ventrally and dorsally. Posteriorly, the arcuate body is supplied by small arteries emanating from the anterior aorta [18]. The subesophageal neuromeres are supplied medially by twelve unpaired transganglionic arteries and laterally by six pairs of arteries which branch off from the leg and pedipalpal arteries. The center of the neuromeres remains largely free of arteries but Huckstorf et al. [18] observed that the neuropils are often supplied by fine arteries which run in tight loops through the subesophageal ganglion.

Similar patterns of intraganglionic arteries have also been observed in Uropygi and Amblypygi (Klußmann-Fricke B.-J., Pomrehn S.W., Wirkner C.S. unpublished data). It can be suggested, then, that extensive supply to the prosomal ganglion is part of the ground pattern of Tetrapulmonata (i.e. Araneae and Pedipalpi), a group that is clearly monophyletic e.g. [19-22]. However, Scorpiones seem to be more closely related to Opiliones [21,22] than to Tetrapulmonata. Considering the similarities between the pattern of intraganglionic arteries in Tetrapulmonata and that in scorpions, it seems likely that a similar system of arterial supply to the nervous system was already present in the arachnid ground pattern.

### Capillaries and *retia mirabilia* in arthropods?

In vertebrates, capillaries are defined as networks of small-diameter vessels (5 – 10 μm) that connect arteries and veins. Other definitions include further structural criteria such as the presence of exclusively endothelial layers and the presence of connective tissue. Functionally speaking, capillaries are defined as the location where the exchange of oxygen and nutrients takes place [10]. Due to the lack of veins in arthropods, the relevant literature defines capillaries in this taxon as a network of arteries connecting afferent and efferent arteries [12,13,18,23]. Since the afferent arteries of the anterior aorta and the small arteries in the prosomal ganglion are directly connected to efferent arteries (e.g. coxal arteries), the vasculature in the prosomal ganglion does represent a form of capillarization. Moreover, Lane et al. [8] state that in scorpions, at least, these channels seem to be lined by endothelial-like layers, making it likely that they are true arteries which are continuous with the rest of the HVS (hemolymph vascular system). In scorpions, then, it does seem possible to talk about capillary networks without flouting the above definitions. Capillary beds connecting afferent and efferent arteries have also, in vertebrates [24] and some echinoderms [25], been termed *retia mirabilia*, though the definitions of this phenomenon vary. In vertebrates a *rete mirabile* is an artery or a vein that branches immediately into a network of small vessels [24] forming numerous anastomoses. If the branches of the network reunite to form an artery again, the phenomenon is known as a bipolar *rete mirabile*[26,27]. In the medical literature, a *rete mirabile* is also defined as the branching of an artery or vein that then reforms into an artery or vein [28]. Examples are the glomeruli malpighii in the human kidney and the portal system in the liver. In other vertebrates, such networks are found in the head arteries of mammals and in the feet of Anseriformes (ducks, geese, swans and their allies) and Spheniscidae (penguins) [29,30], for example. In most of the Anglo-American literature, including the McGraw-Hill “Dictionary of Scientific and Technical Terms” [31], a *rete mirabile* is defined as “a network of small blood vessels that are formed by the branching of a large vessel and that usually reunite into a single trunk; believed to have an oxygen-storing function in certain aquatic fauna”. The definition provided in UBERON [32], an integrative multi-species anatomy ontology, also includes functional aspects, as does that in Wikipedia on which it is based. As we prefer structural definitions of morphological terms (see [1,33,34]), we use the above definitions [26,27] to define the vasculature in the prosomal ganglion of scorpions as a bipolar *rete mirabile* on the grounds that the main arteries branch into capillaries which form numerous anastomoses and, after passing the prosomal ganglion, reunite first into the collection arteries and then into the efferent coxal arteries.

### Supply patterns

The vasculature in the prosomal ganglion of *Brotheas granulatus* corresponds to a high degree with the neuronal organization of the ganglion. The neuropils of the incorporated ganglia are amply supplied by the surrounding arteries, which reticulate on the surface of the neuropils and send loops into them. This is probably to be viewed in the context of the energy requirements of these synapsis-rich structures. Both the somata and the synaptic terminals of nerve cells display a high density of mitochondria and can also be assumed to be involved in a high level of metabolic activity [35]. Thus, the anteroventral regions along the subesophageal midline (i.e. the central ganglion) which exhibit a large number of synaptic contacts and a high level of neuronal activity requiring oxygen and nutrients [13] are also particularly well-supplied. The same applies to the arcuate body [15]. On the other hand, the pectinate neuropils do not seem to be particularly well-supplied, despite the fact that according to Wolf & Harzsch [16], numerous synaptic contacts are found in this area. The degree of capillarization in the CNS of spiders [18] and decapod crustaceans [13] is comparable to that in scorpions and, as in scorpions, certain distinct areas are better-supplied than others. Sandemann [13] reported that the capillary system in the neuropil areas of the optic lobe in *Carcinus maenas* is most elaborate in known synaptic areas. The next best-supplied structures are the somata, while the axons, main nerve bundles and axon chiasmata receive little supply via capillary systems. The same is true of *B. granulatus*, where the synaptic areas and peripherally arranged somata are better supplied than the rest of the nervous tissue, as the arteries reticulate at the border of the neuropils. No distinct supply to the tracts and neurite bundles was observed.

Another suggestion by Sandemann [13] is that dense vascularization could be related to the possible synthetization of transmitter substances in the synaptic terminals. However, as immunohistochemical studies of invertebrate nervous systems show that the somata of immunoreactive neurons also emit a clear signal when transmitter substances are labeled [36], we suggest it is more probable that the numerous mitochondria located in the synaptic contacts need to be supplied intensively with oxygen and nutrients.

In summary, the areas of greatest complexity in capillary networks seems to indicate those areas of the neuropil which require the greatest turnover of fresh hemolymph. This insight and the fact that scorpions have a moderate size and are easy to obtain could be used as the basis for a study into the physiological correlations between oxygen supply and consumption and variations in the neuronal and computational significance of distinct neuropil areas.

### Causes for arterial patterns

As described above, the ganglionic arteries are positioned relatively exactly on the border between the centrally located neuropils and the surrounding somata. This raises the question of why and how this particular pattern becomes established during ontogeny. Unfortunately, little is known about the pattern formation and development of arteries in arthropods, so we can only make suggestions as to the reasons behind the patterns observed in the ganglionic vasculature.

One possible explanation could be that nervous system development actually plays a role in arterial pattern formation. McClendon [37] describes free spaces remaining along the midline of the paired anlagen of adjacent neuromeres in scorpions, into which it is conceivable that the transganglionic arteries grow. The smaller supplying arteries then emanate from the transganglionic arteries and surround the hemi-neuromeres of the ganglia. This, however, is based on the assumption that arteries develop subsequently to the central nervous system anlagen, though it is certainly known from malacostracan crustaceans that the heart only starts to develop once the nervous system anlagen are present (G. Jirikowski, pers. comm.).

Another explanation could be a physiological constraint. Like the tracheae of insects [38], the arteries may grow along an oxygen gradient, forming networks around structures with a high oxygen and/or nutrient demand.

## Conclusions

We could demonstrate that the vascularization in the prosomal ganglion of scorpions is a closed system of capillaries between afferent and efferent arteries. Thus, this capillary network can also be termed a bipolar *rete mirabile*, adopting the structural definition used for vertebrate circulatory systems. However, similar structures have so far only been termed in vertebrates and some echinoderms.

Similar patterns of intraganglionic arteries have also been observed in Uropygi, Amblypygi, and Araneae. Considering these structural correspondences between the pattern of intraganglionic arteries in Tetrapulmonata and that in scorpions, it seems likely that a similar system of arterial supply to the nervous system was already present in the arachnid ground pattern.

Within the *rete mirabile* of the scorpion prosomal ganglion, some regions (i.e. neuropils) are better supplied than others. The areas of greatest complexity in capillary networks seem to indicate those areas of the neuropil which require the greatest turnover of fresh hemolymph. The structural information provided here can now be used in functional neuronal studies to determine the physiological and computational significance of the various neuropils in the complex scorpion nervous system. The fact that scorpions have a moderate size and are easy to obtain could lead to new model organisms for physiological studies on correlations between oxygen supply and consumption and variations in the neuronal and computational significance of distinct neuropil areas.

## Materials & methods

### Materials

Data were gathered from two individuals of the chactid *Brotheas granulatus* in which the injection technique (see below) worked best and provided a complete image of the cerebral vascular system. The taxon sampling also included fourteen buthids, nine vaejovids, four scorpionids, four liochelids, three iurids, two diplocentrids, two chactids, a bothriurid, a chaerilid, a euscorpiid, a pseudochactid and a superstitioniid (see Table 1 in [2]). Scorpion higher classification follows Prendini and Wheeler [39].

### Injection method

In order to prepare injections of the circulatory system, the acrylic casting resin Mercox CL-2R/2B (Vilene Comp. Ltd, Tokyo, Japan) was injected into the heart of ether-anaesthetized specimens using micropipettes (Hilsberg pipettes, diameter 1.0 mm, thickness 0.2 mm; pulled with a KOPF Puller 720). The resin was mixed with approximately 0.05 mg MA initiator prior to injection and placed in a 5 ml syringe which was used to fill the pipettes. The pipettes were placed on an adjustable instrument holder in a mechanical micromanipulator and the tips inserted through the intersegmental membrane between the mesosomal tergites directly into the heart. The specimens were left for several minutes after injecting to allow the resin to polymerize and temper. Specimens destined for MicroCT were fixed in Bouin’s fixative, contrasted with iodine [40] and then critical-point dried (BAL-TEC CPD 030; EMITECH K850). Alternatively, specimens were macerated for 1–2 days by repeated immersion in 10% potassium hydroxide at ambient temperature, followed by washing in a solution of 2 g Pepsin in 10 ml of 2% HCl [41].

### Scanning Electron Microscopy (SEM)

Casts for SEM were air-dried, coated with gold or palladium (BAL-TEC SCD 005; Leica SCD500), and studied using a SEM (LEO 1430; Zeiss DSM 960A), as described by Wirkner and Richter [42]. Corrosion casts were mounted on a specimen holder [43] to achieve a homogeneous black background.

### MicroCT

X-ray imaging was performed with a Phoenix nanotom^®^ (Phoenix|x-ray, GE Sensing & Inspection Technologies) high-resolution MicroCT system in high-resolution mode, using the program *datos|x acquisition* (target: molybdenum, mode: 0–1; performance: ca. 8–13 W; number of projections: 1080–1440; detector timing: 1000–1500 ms; voxel size ca. 2–10 μm). A volume file was generated using the software *datos|x reconstruction* and a stack of virtual sections exported with the software *VGStudio max*. The original μCT image stack of B. granulatus on which most results are based is stored in a public repository (MorphDBase: https://www.morphdbase.de/?B_Klussmann-Fricke_20140304-M-0.1; https://www.morphdbase.de?B_Klussmann-Fricke_20140305-M-1.1)

### 3D Reconstruction

The 3D-reconstruction of the MicroCT-generated virtual sections was carried out using the software Imaris 7.0.0 (Bitplane^©^). A scene was created in the program module “Surpass”, and the volume-rendering function chosen to visualize the entire data set. The contours of the organs studied were marked with polygons on each virtual cross-section using the “Surfaces” function. Different functions (“Isoline” and “Distance”) were used for segmentation. Stacks of polygons were visualized via surface rendering.

### Image processing

Figures were arranged into plates using Corel^®^ Graphics Suite X3. Bitmap images were embedded into Corel Draw X3 files and digitally edited using Coral Photo Paint X3.

## Competing interests

The authors declare that they have no competing interests.

## Authors’ contribution

BJKF and CSW designed the study, carried out resin injections, microCT and SEM imaging. All authors performed 3D reconstructions and analysed the data. BJKF wrote the manuscript with input from CSW. All authors read and approved the final manuscript.
